# Sestrin 2 levels are associated with emphysematous phenotype of COPD

**DOI:** 10.1371/journal.pone.0273652

**Published:** 2022-08-30

**Authors:** Leonidas Angelakis, Andriana I. Papaioannou, Evgenia Papathanasiou, Argiro Mazioti, Maria Kallieri, George Papatheodorou, George Patentalakis, Georgios Hillas, Spyridon Papiris, Nikolaos Koulouris, Stelios Loukides, Petros Bakakos

**Affiliations:** 1 1^st^ University Department of Respiratory Medicine, National and Kapodistrian University of Athens, Athens, Greece; 2 2^nd^ University Department of Respiratory Medicine, National and Kapodistrian University of Athens, Athens, Greece; 3 Radiology Department, “Mediterraneo” Hospital, Athens, Greece; 4 Research Laboratory, «401» General Military Hospital of Athens, Athens, Greece; 5 5^th^ Respiratory Medicine Department, “Sotiria” Hospital, Athens, Greece; Hualien Tzu Chi Hospital, TAIWAN

## Abstract

Sestrins (Sesns) are a family of highly conserved stress-inducible proteins and various stresses have been shown to strongly up-regulate them. Sestrin 2 (Sesn2) deficiency has been shown to partially suppress pulmonary emphysema. The aim of this study was to evaluate Sesn2 levels in COPD patients and its possible associations with the presence of emphysema and blood eosinophils. All patients underwent lung function testing and high-resolution computed tomography (HRCT) of the chest. The presence of emphysematous lesions in >15% of the pulmonary parenchyma was considered as significant emphysema. Sixty-seven patients were included in the study. 40/67 patients were characterized as having significant emphysema. Patients with significant emphysema had higher levels of Sesn2 (ng/ml) [median (IQR) 6.7 (2.7,10.3 vs 1.09 (0.9,1.9), p<0.001)] and significantly lower % and absolute blood eosinophil counts (cells/μL) compared to patients without emphysema [1 (0, 2) vs 4 (2, 4) p<0.001 and 62 (0, 110) vs 248 (180, 300), p<0.001 respectively]. Sesn2 presented a significant positive correlation to the score of emphysema in HRCT (r_s_ = 0.87, p<0.001) and similar positive but weaker correlation to FRC (r_s_ = 0.27, p = 0.024). Negative correlations were observed between Sesn2 and either the % of blood eosinophils and/or the absolute blood eosinophil count (r_s_ = -0.79, p<0.001, and r_s_ = -0.78, p<0.001 respectively). Sesn2 levels above 1.87 ng/ml showed a high diagnostic performance for the presence of significant emphysema in HRCT with an AUC 0.93, 95% CI (0.85,0.98), p<0.001. Sesn2 could serve as a potential biomarker of emphysema.

## Introduction

Chronic obstructive pulmonary disease (COPD) presents with persistent respiratory symptoms (such as cough, sputum production and dyspnea) and airflow limitation that is attributed to airway and/or alveolar abnormalities [1] Emphysema represents the most well recognized anatomic change in the lung parenchyma of COPD patients [2]. High resolution computed tomography (HRCT) has proven to be a reliable tool for the evaluation of structural changes within the lung caused by a variety of respiratory diseases including COPD [3]. Two distinct types of lung changes occur in COPD patients, airway remodeling and emphysema. Airway remodeling can be partly reversible while emphysema correlates with destroyed lung tissue and is considered irreversible. HRCT imaging can discriminate COPD patients into emphysema-dominant and airway-dominant phenotype [4].

Sestrins (Sesns) consist of a family of highly conserved stress-inducible proteins and various stresses such as hypoxia, oxidative stress and DNA damage seem to up-regulate them [5]. Sestrin 2 (Sesn2) is thought to reduce oxidative stress [6–9]. First by rescuing the peroxidase activity of overoxidized peroxiredoxins and secondly by activating the transcription factor NRF2 (nuclear factor erythroid 2-related factor 2), which is a potent antioxidant gene inducer. It has been reported that although sestrins are molecules which attenuate ageing and suppress development of many age-related diseases such as myocardial infarction, muscle atrophy, diabetes, and immune dysfunction, they have an probable deleterious role in the development of COPD [10]. In mouse models of COPD, Sesn2 knock down resulted in suppression of the development of emphysema while Sesn2 null animals were less susceptible to the development of COPD when exposed to cigarette smoke [9]. Furthermore, it has been reported that Sesn2 is accumulated in COPD smokers indicating an association between Sesn2 expression and COPD development [7].

The aim of the present study was to examine circulating Sesn2 levels in COPD patients and examine possible correlations between Sesn2 levels and the extent of emphysema as estimated using HRCT. As secondary outcomes we further examined whether Sesn2 levels are associated to lung function parameters and blood eosinophils.

## Methods

### Study design

In the present cross-sectional observational study, we have included consecutive patients with COPD according to the Global Initiative for Chronic Obstructive Lung Disease (GOLD) recommendations [1] which were treated in the outpatient clinics of the 1^st^ and 2^nd^ University Respiratory Medicine Departments (“Sotiria” and “Attikon” University Hospital). Inclusion criteria were:

- Previous COPD diagnosis
- Treatment for stable COPD for at least 6 weeks prior to the inclusion to the study
- Ability of the patient to perform a spirometry test
- Acceptance of the patient to participate to the study
- Exclusion criteria were:
- Significant respiratory disease other than COPD, i.e coexistence of asthma
- Significant uncontrolled cardiovascular disease
- Current Malignancy or history of malignant disease
- Presence of eosinophilic lung diseases
- Previous (last 8 weeks) CODP exacerbation
- Treatment with systemic corticosteroids or antibiotics for any reason during the last 8 weeks
- Chronic respiratory failure requiring long term oxygen therapy
- Inability or unwillingness of the patient to collaborate with the study investigators

Demographic characteristics of the patients, including age, sex, BMI (expressed in kg/m^2^), comorbidities and smoking habit were recorded. All patients underwent physical examination, pulse oxymetry, and pulmonary function tests. The diagnosis and severity of COPD was established by post-bronchodilator spirometry on stable condition according to GOLD recommendations. Patients’ treatment including inhaled corticosteroids was also recorded. Peripheral blood samples were collected from all patients and white blood cell and blood eosinophil counts were recorded. Pulmonary function tests and blood samples collection were always performed in the morning. The study protocol was approved by the local Ethics Committee (Scientific Board of Sotiria Hospital, approval no 3747/16-2-2015) and all patients provided written informed consent.

### Lung function

Pulmonary function tests (PFTs) were performed using a commercially available system (Master Screen, Erich Jaeger GmbH, Wuerzburg, Germany) Forced vital capacity (FVC), forced expiratory volume in one second (FEV_1_), FEV_1_/FVC ratio, and forced expiratory flow 25–75 (FEF_25-75%_) were recorded. COPD severity was evaluated by post-bronchodilator values (i.e. 30 minutes after the administration of 400 mg salbutamol with a spacer) according to GOLD recommendations. [1]. The single breath method was used for the assessment of the diffusing capacity for carbon monoxide (DL_CO_) with the patient in the sitting position [11]. Lung function measurements were expressed as percentages of predicted values and were performed according to the American Thoracic Society (ATS) guidelines [12].

### HRCT

All patients underwent HRCT using either a Somaton HiQ or a Somaton Plus scanner (Siemens, Erlanger, Germany). Scans were performed with 1–1.5mm section thickness and a 1–2 sec scanning time during breath holding at end inspiration. Films were assessed by two experienced radiologists in HRCT who were blinded to the functional, laboratory and clinical data of the patients. The degree of emphysema was calculated using a visual emphysema score [13]. Emphysema score was measured visually, as areas of low attenuation that contrast with the normal attenuation of surrounding lung parenchyma. A threshold HU value, as described in quantitative CT (QCT emphysema is defined as ≥ 5% of lung volume occupied by low attenuation areas ≤− 950 Hounsfield units) was not applicable. All HRCT images in inspiration were evaluated and scored and the sum was divided by the slices, in order to measure total emphysema score. Briefly, emphysema was identified as areas of hypovascular low attenuation and was graded with a five-point scale taking into consideration the percentage of lung involved as follows: 0: no emphysema; 1: up to 25% of the lung parenchyma involved; 2: between 26–50% of lung parenchyma involved; 3: between 51–75% of the lung parenchyma involved; and 4 between 76–100% of lung parenchyma involved. Grades of the axial images of each lung were added and divided by the number of images evaluated to yield emphysema scores that ranged from 0 to 4 [13]. The scores of the two radiologists were added and divided by two and the mean value was used for the analysis. Emphysematous phenotype was defined by the presence of emphysematous lesions ≥15% of the pulmonary parenchyma (i.e. score ≥0.6) [14].

### Sesn2 levels measurement

Peripheral blood samples were collected from each patient. Serum supernatant was collected after centrifugation at 3,500 x g for 10 min and stored at –80°C until measurement. Sesn2 levels were assayed by an Enzyme-linked Immunosorbent Assay Kit for Sesn2 (Cloud-Clone Corp., USA) according to the manufacturer’s instructions.

### Statistical analysis

Categorical variables were presented as n (%), whereas numerical variables were presented as mean±SD or median (interquartile ranges) for normally distributed and skewed data respectively. Normality of distributions was checked using Kolmogorov-Smirnov test. Comparisons between groups were performed using chi-square tests for categorical data, as well as Mann-Whitney U-tests for numerical data, since the distribution was skewed. Correlations were performed using Spearman correlation coefficient. For the assessment of the diagnostic performance of Sesn2 for significant emphysema in HRCT, a receiver operating characteristic (ROC) curve was created. Area under the ROC curve (AUC) with 95% confidence intervals (CI) and their differences from 0.5 was calculated. Sensitivity, specificity, positive (PPV) and negative predictive value (NPV) were calculated for the optimal cut-off value. All p-values <0.05 were considered statistically significant. Data were analysed using SPSS 17.0 for Windows (SPSS Inc., Chicago, IL, USA) and Graphs have been created using GraphPad Prism 6 (GraphPad Software, Inc La Jolla CA USA).

## Results

Sixty-seven consecutive patients with stable COPD were included in the study. Demographic, functional and inflammatory characteristics of the study participants are shown in Table 1.

**Table 1:** Demographic, functional and inflammatory characteristics of the study participants (all and according to the presence of emphysema).

Variable	All	Emphysema	Non-emphysema	p value
	N = 67	N = 40	N = 27	
**Age (years)**	68 (61, 71)	69(62,73)	65(54,70)	0.071
**Sex (male) n(%)**	55 (74)	32(80)	23(85)	0.187
**BMI kg/m** ^**2**^	26(23,30)	25(22,29)	27(24,30)	0.062
**Current smokers/ Ex-smokers**	42/25	26/14	16/11	0.456
**n (%)**	(63/37)	(65/35)	(59/41)	
**Pack years**	65 (45–88)	60(43–80)	69(48–90)	0.104
**FEV**_**1**_ **(%pred.)**	51±18	49±18	54±17	0.136
**FEV1 (L)**	1.40±0.56	1.35±0.58	1.49±0.56	
**FEV**_**1**_ **/FVC**	53±11	51±12	56±10	0.061
**FRC % pred**	123 (91–154)	138±46	117±41	**0.036**
**DLCO %pred**	49±14	46±14	53±14	0.053
**ICS use, n (%)**	33 (49.2)	15(37.5)	18(66)	**<0.001**
**Exacerbations in the previous year (n)**	1.0 (0.0,1.0)	1(0,1)	0(0,1)	0.286
**Sesn2 ng/mL**	2,45 (0.91,8.9)	6.7 (2.7,10.9)	1.09 (0.9,1.9)	**<0.001**
**Emphysema score on HRCT**	1.1(0.4,2.7)	2.25(1.42,3)	0.4(0.3,0.5)	**<0.001**
**Blood Eosinophils %**	2(1–3)	1(0,2)	4(2,4)	**<0.001**
**Blood eosinophils absolute count (cells/μL)**	110(62–190)	62(0,110)	248(180,300)	**<0.001**
**Metabolic disease n (%)**	5(7.5)	2(5)	3(11)	0.350

Values are presented as mean (SD) or Median (IQR) for normally and skewed variables respectively, unless otherwise indicated. Abbreviations: BMI: Body mass index, DLCO: Diffusion Lung Capacity, FEV_1_: Forced Exhaled Volume in one second, FVC: Forced Exhaled Vital Capacity, FRC: Functional residual capacity, ICS: Inhaled Corticosteroids, Sesn2: Sestrin 2, HRCT: High Resolution Computed Tomography.

### Sesn2 levels according to the presence of significant emphysema

**Fig 1:**
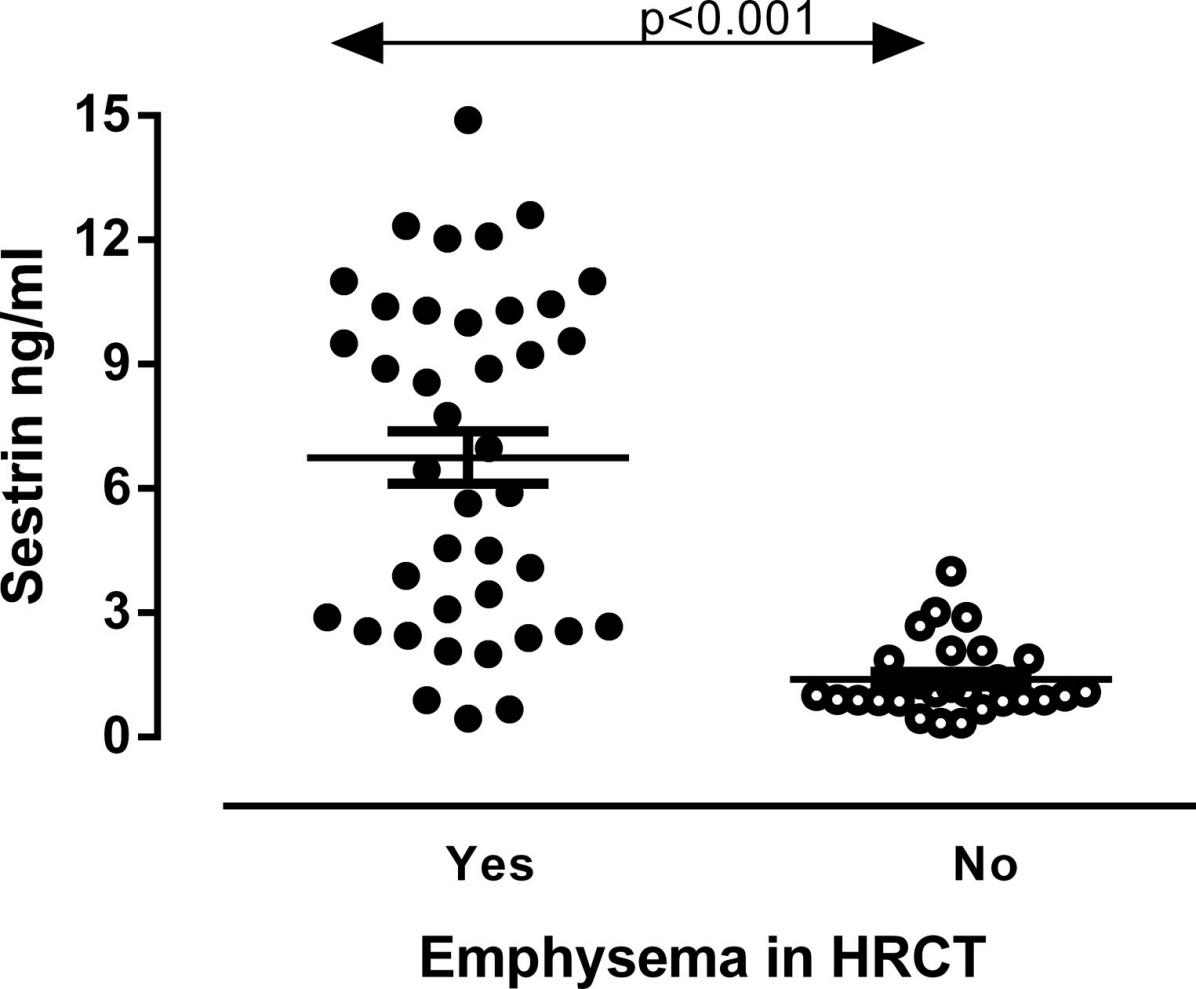
Serum Sesn2 levels according to the presence of emphysema in HRCT. For data see text. Abbreviations: HRCT: High Resolution Computed Tomography.

Patients with significant emphysema, as defined by the presence of emphysematous lesions in ≥15% of the pulmonary parenchyma had higher levels of Sesn2 (ng/ml) compared to those with emphysematous lesions in <15% of the pulmonary parenchyma [6.7 (2.7,10.3) vs 1.09 (0.9,1.9), p<0.001, Fig 1]. Patients with significant emphysema had significantly lower % and absolute blood eosinophil counts, higher levels of FRC compared to patients without emphysema. (Table 1). Sesn2 levels(ng/ml) did not differ between current and ex-smokers (2,67 (0,97,9,3) vs 2.07 (0,89,7,39) p = 0.442).

### Correlations of Sesn2 with study parameters

**Fig 2:**
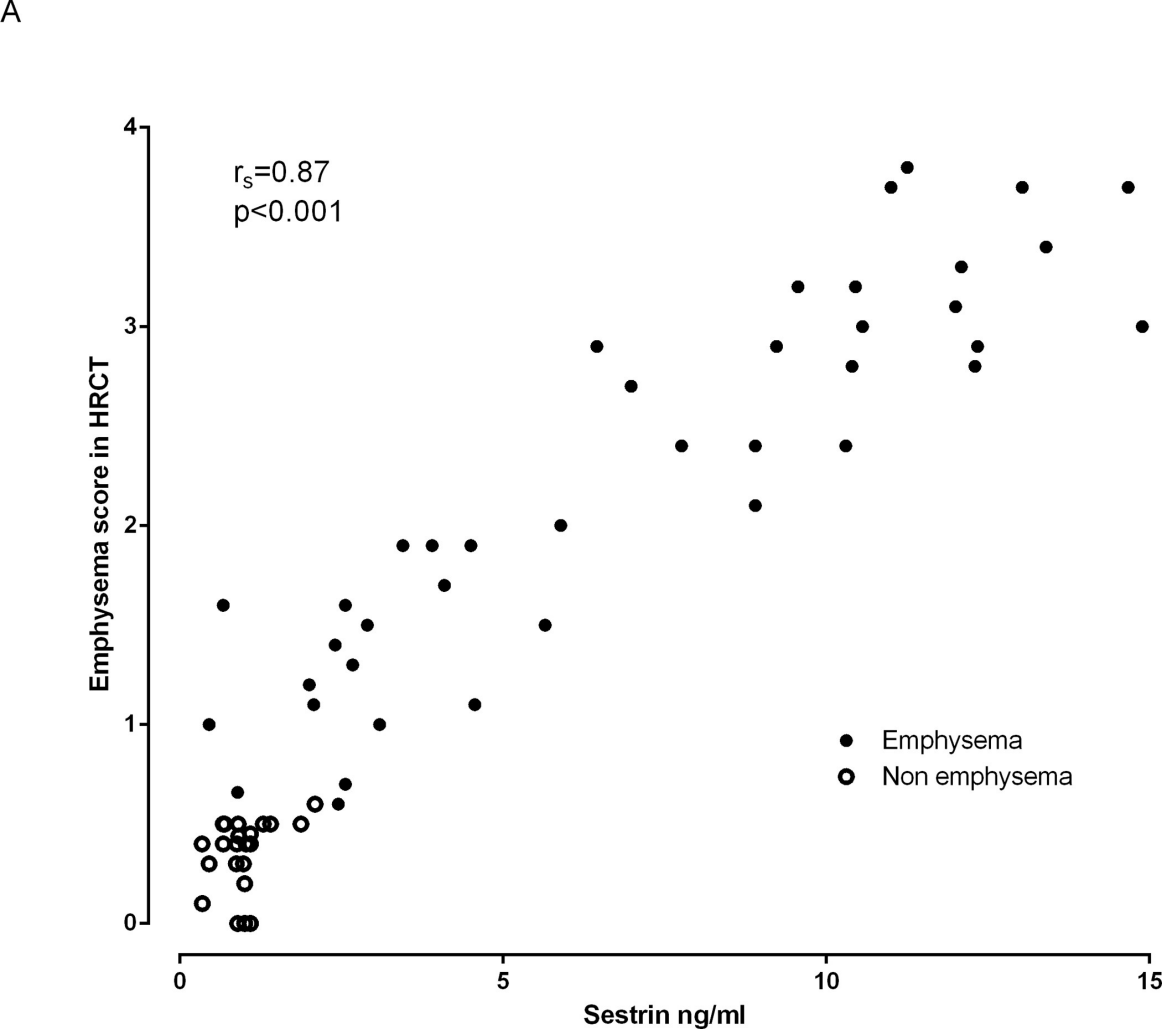
Correlation between Sesn2 and emphysema score in HRCT. For data see text. Abbreviations: HRCT: High Resolution Computed Tomography.

**Fig 3:**
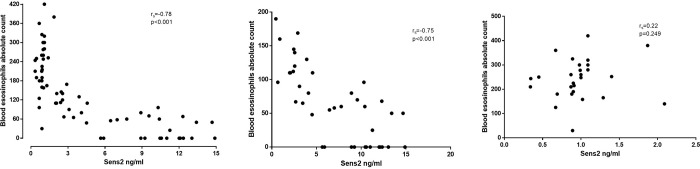
Correlation between Sesn 2 and absolute blood eosinophil number A: All patients, B: patients with emphysema C: Patients without emphysema.

Table 2 summarizes the major correlation data either for the whole group or/and for the two subgroups (emphysematous lesions in ≥15%, emphysematous lesions in <15%). Sesn2 presented a significant positive correlation to the score of emphysema in HRCT (r_s_ = 0.87, p<0.001, Fig 2). Similar positive but weaker correlation was observed between Sesn2 and FRC (r_s_ = 0.27, p = 0.024) and even weaker but negative between Sesn2 and DLCO (r_s_ = -0.24, p = 0.047). Negative correlations were observed between Sesn2 and either the % of blood eosinophils or/and the blood eosinophils absolute count (r_s_ = -0.79, p<0.001, and r_s_ = -0.78, p<0.001 respectively). In those with >15% emphysema a highly significant correlation between Sesn2 and eosinophils either as percentage or as an absolute count was observed (r_s_ = -0.69, p<0.001, and r_s_ = -0.75, p<0.001 respectively). However, in the non-emphysema group no correlation between Sesn2 and either the % of blood eosinophils or/and the blood eosinophils absolute count was observed (r_s_ = -0.26, p = 0.182, and r_s_ = -0.22, p = 0.249 respectively). Correlations between the levels of Sesn2 and the absolute number of blood eosinophils are shown on Fig 3A–3C. No other significant correlation was observed.

**Table 2:** Major correlation data for Sesn2 levels (ng/ml) either for the whole group or/and for the two subgroups (emphysematous lesions in ≥15%, emphysematous lesions in <15%).

Variable	All	Emphysema	Non emphysema
	N = 67	N = 40	N = 27
FEV_1_ (%pred.)	r_s_ = -0.17, p = 0.162	r_s_ = -0.11, p = 0.496	r_s_ = -0.04, p = 0.818
FEV_1_/FVC	r_s_ = -0.16, p = 0.195	r_s_ = -0.13, p = 0.413	r_s_ = -0.14, p = 0.455
FRC % pred	**r**_**s**_ **= 0.27, p = 0.024**	**r**_**s**_ **= 0.24, p = 0.038**	r_s_ = -0.01, p = 0.478
DLCO %pred	**r**_**s**_ **= -0.24, p = 0.047**	r_s_ = -0.18, p = 0.257	r_s_ = 0.04, p = 0.827
Emphysema score on HRCT	**r**_**s**_ **= 0.87, p<0.001**	**r**_**s**_ **= 0.90, p<0.001**	r_s_ = 0.21, p = 0.314
Blood Eosinophils %	**r**_**s**_ **= -0.79, p<0.001**	**r**_**s**_ **= -0.69, p<0.001**	r_s_ = 0.26, p = 0.182
Blood eosinophils absolute count (cells/μL)	**r**_**s**_ **= -0.78, p<0.001**	**r**_**s**_ **= -0.75, p<0.001**	r_s_ = 0.22 p = 0.249

Bold letters indicate statistical significance. Abbreviations DLCO: Diffusion Lung Capacity, FEV_1_: Forced Exhaled Volume in one second, FVC: Forced Exhaled Vital Capacity, FRC: Functional residual capacity, HRCT: High Resolution Computed Tomography.

### Diagnostic performance of Sesn2 for the presence of significant emphysema in HRCT

**Fig 4:**
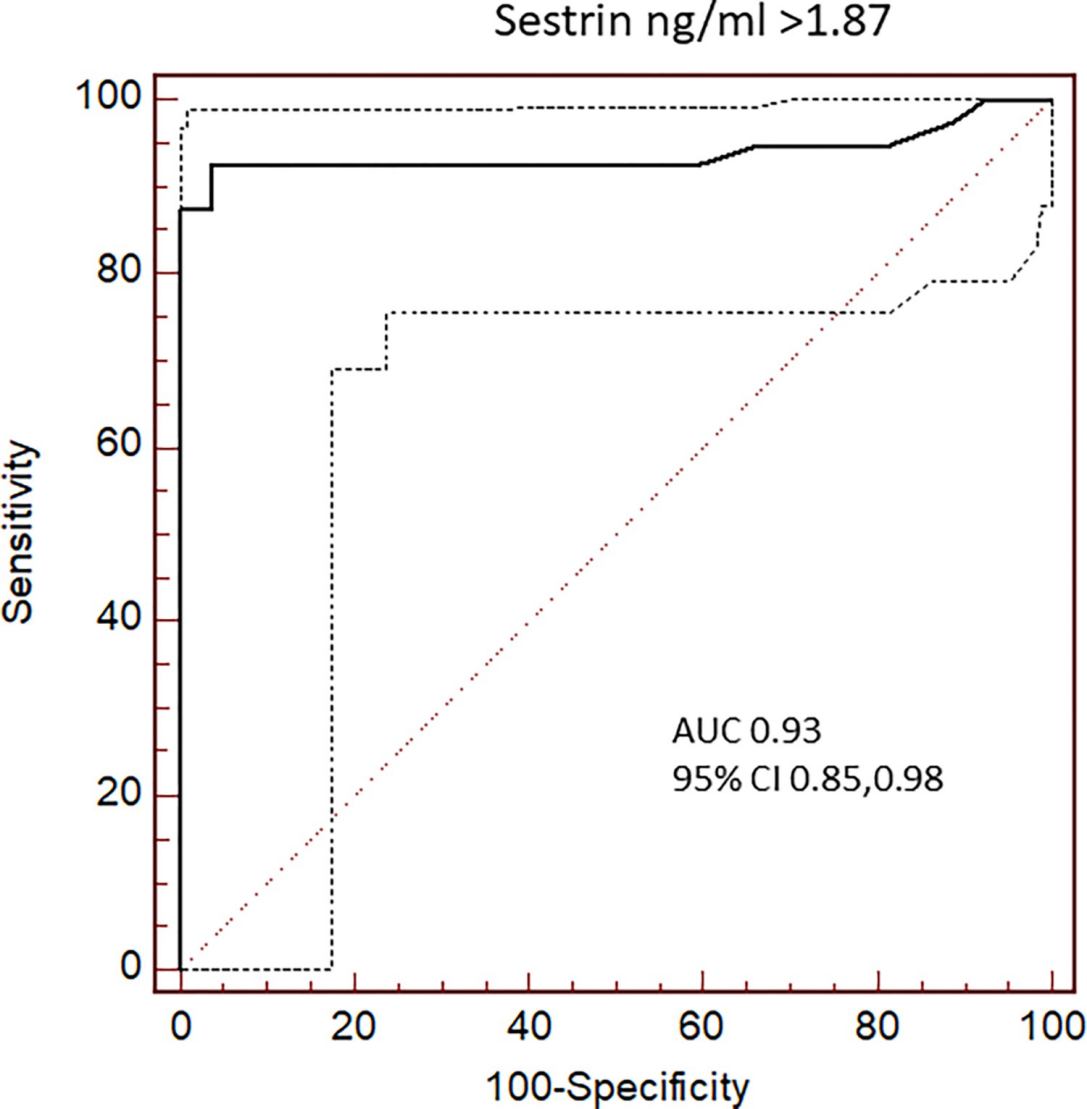
Diagnostic performance of Sesn2 for the presence of significant emphysema in High Resolution Computed Tomography (HRCT). For data see text.

Sens 2 levels were a significant predictor of emphysema AUC 0.93, 95% CI (0.85,0.98), p<0.001. Sesn2 levels above 1.87 ng/ml showed a high diagnostic performance for the presence of significant emphysema in HRCT with a 92.5% sensitivity and 96.3% specificity PPV = 100% and NPV = 65,5%. Fig 4.

## Discussion

In the present cross-sectional study, we have shown that patients with significant emphysema in HRCT present with significant higher levels of Sesn2 compared to those without emphysema. Additionally, Sesn2 presented a positive correlation to the score of emphysema in HRCT and a negative correlation to DLCO. Finally, Sesn2 was negatively associated to either the % of blood eosinophils or/and the blood eosinophils absolute count.

This is the first study to evaluate Sesn2 levels among COPD patients according to the presence of emphysema. Previous studies [5–9] have shown that Sesn2 inactivation leads to ROS accumulation and oxidative stress, that plays a role in the pathogenesis of COPD. Moreover, ROS are crucial regulators of signal transduction pathways, such as platelet-derived growth factor receptor β (PDGFRβ) and its signaling has been shown to be induced by ROS accumulating [7]. On the other hand, the TGF-β signaling that partly encodes elastin but not the profibrotic genes collagen I and collagen III was selectively up-regulated by the inactivation of Sesn2 in Ltbp4S–/–mice through a ROS independent pathway [7, 9].

It has been shown that the lack and/or excessive degradation of elastin enhance the emphysema progress. Using a pulmonary emphysema model induced by inactivating mutation of the small splice variant of the Ltbp4 gene (Ltbp4S–/–), Frank et al. demonstrated that the Sesn2 null alleles (Ltbp4S–/–Sesn2–/–) significantly reduced the pulmonary emphysema of Ltbp4S–/–mice, as Ltbp4S–/–Sesn2–/–mice presented less parenchymal lesions and lung compliance. Sesn2 may also inhibit PDGFRβ expression and in another study, it was demonstrated that the development of cigarette-smoke-induced pulmonary emphysema was prevented by the mutational inactivation of Sesn2 through the up regulation of PDGFRβ expression [7, 9]. These studies conclusively support a role for Sesn2 in the development of pulmonary emphysema and are in accordance with our findings showing that emphysematous patients are characterized by higher levels of Sesn2. Finally, although emphysema is a radiological diagnosis the increased levels of Sesn2 in emphysematous patients in combination of the high predictive value of this biomarker for the presence of significant emphysema, support the hypothesis that Sesn2 is playing an adverse role in the development of emphysematous lesions [10].

In a previous study [15] has been reported that patients with significant emphysema in HRCT present lower levels of blood eosinophils while the study of Singh et al. [16], which included patients from the ECLIPSE cohort, showed that the progression in the emphysematous lesions was enhanced in subjects with persistent eosinophil counts <2%. These observations are in accordance with our findings showing lower blood eosinophil levels in patients with emphysema, while the presence of negative correlations between Sesn2 and either the % of blood eosinophils or the absolute blood eosinophil count also shows that Sesn2 levels are also related to the presence of emphysematous lesions. Accordingly, one would postulate a role for Sesn2 in the suppression of eosinophilic inflammation in COPD. To our knowledge, currently there are no data connecting Sesn2 with peripheral blood eosinophils. The association between Sesn2 and eosinophils detected in our study might not be causal but simply an indirect one. These correlations between Sesn2 and eosinophils were not observed in the non-emphysema group indicating that this association is indirect, and possibly mainly related to the presence of emphysema. Furthermore, we cannot exclude the possibility that Sesn2 levels were affected by the use of ICS since patients with emphysema used significantly less ICS compared to non-emphysema COPD patients although further studies are needed to evaluate the possible effect of ICS in Sesn2 levels in COPD patients.

Furthermore, the observation that blood eosinophil level was greater in COPD subjects without emphysema, line with increasing evidence that blood eosinophils might have a beneficial, rather than a detrimental, effect in COPD [17, 18]. This finding is of interest considering that therapies geared to reduce eosinophils in COPD had no beneficial effects [19, 20]. Our findings indicate that Sesn2 might serve as a biomarker for emphysema among COPD patients and may also be a candidate biomarker for future treatments that aim at reducing emphysema. Its correlation–though weak- with FRC supports the well-known association of static hyperinflation with COPD and mainly emphysema. We expected a more significant association between Sesn2 and DLCO as the latter is a sensitive marker of emphysema. The small number of patients that comprised our study group as well as the use of a HRCT visual scoring for emphysema might explain the lack of a highly significant association.

Our study has some limitations. First, this is a cross-sectional observational study without the inclusion of a healthy control group while a second group of patients was not used in order to validate our results. Secondly, the quantification of emphysema in HRCT was performed through an observational method, instead of dedicated CT software. However, this method is easy to use in clinical practice and presents excellent correlation with densitometry quantitation. Furthermore, to minimize the potential bias by human rating, both radiologists which scored the emphysematous lesions were blinded to the functional, laboratory and clinical data of the patients. We must also admit that Sesn2 is also elevated in several diseases such as obstructive sleep apnea, atherosclerosis and cardiovascular disease which are common comorbidities in COPD patients [21, 22]. Moreover, the lack of data regarding the time of first COPD diagnosis and duration of COPD may have influenced our findings. Finally, blood eosinophil count may vary from time to time in COPD and a single test is not safe to definitely define whether patients with emphysema had persistently low absolute eosinophil counts.

In conclusion, we have shown that patients with significant emphysema present higher levels of Sesn2 and these levels correlate to the score of emphysema in HRCT. This finding may imply that Sesn2 has an enhancing effect on the development of pulmonary emphysema and could serve as a potential biomarker of COPD and mainly emphysema. Accordingly, patients with emphysema might benefit from treatment with antagonists of Sesn2.

## Supporting information

S1 Data (XLSX)
